# Septal Pacing of Right Ventricle: Has The Last Word Been Said?

**DOI:** 10.1016/s0972-6292(16)30730-6

**Published:** 2014-03-12

**Authors:** Hygriv B Rao

**Affiliations:** Senior Consultant Cardiologist and Electrophysiologist, KIMS Hospitals, Secunderabad, India

**Keywords:** Right Ventricule, Septal Pacing

 The deleterious effects of Right ventricular (RV) pacing on development of ventricular dyssynchrony leading to heart failure, genesis of atrial fibrillation and consequent increased mortality have been repeatedly demonstrated in numerous pacing studies. Trials with dual chamber pacemakers and defibrillators did not succeed in negating these effects, raising the question if it was RV pacing per se or RV apical pacing that was the culprit. Innovations in algorithms to reduce RV pacing were simultaneously supplemented by investigation into alternative sites of pacing to counter these undesirable outcomes. These included pacing of the His bundle, right ventricular outflow tract (RVOT), mid and apical septum. Dictated by the ability to achieve satisfactory lead stability and thresholds, the search has practically narrowed to septal pacing. Physiologically septal pacing results in earlier depolarization of the septum and a closer conduction sequence to normal. This would be expected to reduce LV activation time, prevent dyssynchrony and preserve LV function. RVOT remains the most investigated non- apical pacing site, though other septal sites theoretically can be expected to result in a similar benefit by achieving a narrow QRS and a physiological ventricular activation.

 Studies in the real world however found that the theoretical expectations of septal pacing did not automatically translate into consistent clinical benefits. Several hypotheses can be offered to explain this discrepancy. First pertains to the complexity of the RVOT anatomy which encompasses septal, anterior and free wall segments. Positioning the ventricular lead in each of these areas may not be in operator’s control and more importantly all these areas cannot be assumed to be similar in terms of pacing outcomes. Higher septal areas may be closer to aorta than LV and pacing infundibular region may result in higher thresholds. In fact it was found that only a third of RVOT pacing positions truly achieved a septal position using the conventional fluoroscopic views. Secondly, even when the lead is truly septal, in practice it is not always feasible to achieve a precise pacing target in the septum. Also, there exists no concrete data on the differential outcomes of different septal positions from high to low, making it difficult to advocate the superiority of one over the other. Thus, there exists considerable expected heterogeneity with respect to septal pacing. The third aspect concerns the imaging and electrocardiographic guidance of the septal positioning of the pacing lead. Typically three fluoroscopic views are recommended- the AP, LAO and RAO to ensure septal positioning and avoiding the coronary sinus ostium. Yet one should appreciate that conventional fluoroscopic guidance for achieving targeted lead positions is not infallible. Surface ECG demonstrates a narrow QRS complex when paced from any of the areas of septum and a negative QRS in lead I, is indicative of anterior rather than septal position. This fact has however not been validated by clinical studies and there is no uniform agreement even when 3 dimensional electroanatomical mapping studies are used. Lastly, there is a lack of clarity on the duration of time required to perceive the beneficial effects of septal pacing and varied follow up times in studies involving septal pacing contribute to conflicting results.

 It follows that reliable comparative assessment of implant parameters, procedural complications, cardiovascular and mortality endpoints amongst different septal areas will be possible only if there is standardization of lead position in the septum. In addition, it is necessary to evolve an objective imaging or ECG parameter to authenticate the lead position. It is envisaged that studies with targeted septal pacing locations will provide the much awaited clinical evidence, applicable to a broad population. Three such studies were designed, with specified lead locations– Protect pace (High septum), RASP (RV Inflow), and Optimize RV (Mid septum), the last of which was abandoned. The ongoing trials on non-apical pacing by protocol have unambiguous lead positions, with a fair follow up time and it is expected that their results will provide further insight. A more important fact that such studies are expected to unravel is, if it is a worthwhile effort for the operators to go through the learning curve and challenges to master the technique of targeted septal pacing.

